# Predictors of interstitial lung disease in early systemic sclerosis: a prospective longitudinal study of the GENISOS cohort

**DOI:** 10.1186/ar3125

**Published:** 2010-09-02

**Authors:** Shervin Assassi, Roozbeh Sharif, Robert E Lasky, Terry A McNearney, Rosa M Estrada-Y-Martin, Hilda Draeger, Deepthi K Nair, Marvin J Fritzler, John D Reveille, Frank C Arnett, Maureen D Mayes

**Affiliations:** 1 Division of Rheumatology and Clinical Immunogenetics, University of Texas-Houston, 6431 Fannin, Houston, TX 77030, USA; 2 Center for Clinical Research and Evidence-Based Medicine, University of Texas-Houston, 6431 Fannin, Houston, TX 77030, USA; 3 University of Texas Medical Branch at Galveston, 301 University Boulevard, Galveston, TX 77555, USA; 4 Division of Pulmonary, University of Texas - Houston, 6431 Fannin, Houston, TX 77030, USA; 5 University of Texas-San Antonio, 4502 Medical Drive, San Antonio, TX 78229, USA; 6 University of Calgary, 3330 Hospital Drive NW, Calgary, Alberta T2N 4N1, Canada

## Abstract

**Introduction:**

The objective of the present study was to examine the association of baseline demographic and clinical characteristics with sequentially obtained measurements of forced vital capacity (FVC), expressed as a percentage of the predicted value, and to identify predictors of the decline rate in FVC over time in the Genetics versus Environment in Scleroderma Outcome Study (GENISOS).

**Methods:**

To date, 266 patients have been enrolled in GENISOS, a prospective, observational cohort of patients with early systemic sclerosis. In addition to pulmonary function tests (PFTs), clinical and laboratory data were obtained from each patient. We analyzed 926 FVC measurements utilizing generalized linear mixed models. The predictive significance of baseline variables for the decline rate in FVC was investigated by the interaction term between the variable and the follow-up time within the first 3 years after enrollment as well as throughout the entire follow-up time.

**Results:**

The cohort consisted of 125 white, 54 African American, and 77 Hispanic patients with average disease duration of 2.5 years at enrollment. The mean follow-up time was 3.8 years, ranging up to 11.4 years. A number of baseline variables, including antibody status, African American ethnicity, disease type, baseline PFT values, modified Rodnan Skin Score, fibrosis on chest radiograph, and lung and skin subscores of the Severity Index, were associated with serially measured FVC levels. However, only the presence of anti-topoisomerase I antibodies (ATA) was associated with lower FVC levels (*P *< 0.001) as well as accelerated decline rate in FVC within the first 3 years of follow-up (*P *= 0.02). None of the baseline variables predicted the rate of decline in FVC on long-term follow-up. Patients with rapidly progressive ILD, however, were under-represented in the long-term follow-up group because the accelerated rate of decline in FVC was associated with poor survival (*P *= 0.001).

**Conclusions:**

Presence of ATA was the only baseline variable associated with differential FVC levels, predicting the rate of decline in FVC within the first 3 years of follow-up. The association of faster decline in FVC with poor survival further emphasizes the need for identification of predictive biomarkers by collection of genetic information and serial blood samples in cohort studies.

## Introduction

Systemic sclerosis (SSc) is associated with substantial morbidity and mortality [1,2], and leads to detrimental effects on health-related quality of life [3]. Pulmonary involvement, including both interstitial lung disease (ILD) and pulmonary arterial hypertension, has become the primary cause of SSc-related death [4]. Although a variety of pulmonary function test (PFT) measures have long been used to study ILD in SSc, only the forced vital capacity (FVC) has been validated as an outcome measure in randomized controlled trials [5]. Furthermore, the Genetics versus Environment in Scleroderma Outcome Study (GENISOS) confirmed previous observations [6,7] that FVC <50% at baseline was highly predictive of mortality in SSc [2].

Sequential measurements of pulmonary function in patients with SSc have shown remarkable variability in the progression of restrictive lung disease, ranging from an indolent course with stable PFT values to a rapidly progressive disease leading to respiratory failure and eventually death [7-10]. Anti-topoisomerase I antibodies (ATA) and the absence of anti-centromere antibodies (ACA) are associated with the presence of ILD at baseline [11-15]. However, the data on their predictive role for the decline in FVC percentage predicted over time are conflicting. While ACA and ATA were not predictive of the FVC decline rate in some studies [7,14,16], ATA and diffuse cutaneous SSc were predictive of the FVC decline rate in the univariate analysis in one study; however, only diffuse cutaneous involvement was predictive of a decrease in FVC in the multivariate analysis in this study [17]. Similarly, other demographic and clinical features associated with ILD at the baseline visit - such as male sex [7], African American ethnicity [7,15] and cardiac involvement [7,18] - did not predict the rate of decline in percentage predicted FVC [7,16,17,19-21]. Studies investigating the predictive significance of baseline percentage predicted FVC have reported inconsistent results. Low baseline FVC was associated with an accelerated decline in pulmonary function in some reports [16,22], but not in other studies [7,9,14,19-21,23].

Discrepant results may be explained by methodological differences including retrospective study design [7,12,14,16,22,23], patient selection for clinical trials [20,21] and small sample size [8,9,19].

Previously we reported the association of demographic and clinical characteristics with pulmonary disease at baseline in the GENISOS cohort. African American ethnicity, presence of cardiac involvement, modified Rodnan Skin Score [24], serum creatine phosphokinase levels and absence of ACA were negatively related to percentage predicted FVC at the baseline visit [18].

The objective of the present prospective study was to examine the association of baseline variables with sequentially obtained percentage predicted FVC and to identify baseline demographic and clinical predictors of the decline rate in percentage predicted FVC over time. Such a predictive model would be highly valuable both in clinical practice and in the design of clinical trials.

## Materials and methods

GENISOS was designed to identify factors predictive of various outcomes in SSc. This prospective cohort study is a collaborative effort between the University of Texas Health Science Center at Houston, the University of Texas Medical Branch at Galveston and the University of Texas Health Science Center at San Antonio. Recruitment into the study started in 1998. Initial enrollment and follow-up study visits are ongoing.

### Patient selection

Details of patient selection and recruitment have been previously published by Reveille and colleagues [25]. Patients who fulfilled the following inclusion criteria are enrolled: age ≥18 years; diagnosis according to the American College of Rheumatology (formerly the American Rheumatism Association) preliminary classification criteria for SSc [26]; disease onset within the previous 5 years (defined as the first non-Raynaud's phenomenon symptom); and defined ethnicity with all four grandparents from the same ethnic group. Patients who had SSc-like illnesses associated with environmental, ingested, or injected agents were excluded from the study.

All enrolled patients at the time of analysis were included in the current study. Patients were assigned to the following four ethnic groups: white, African American, Hispanic and other. A total of 10 patients were assigned to the other group consisting of one Native American and nine Asian patients. The study was approved by the institutional review board of all participating sites, and written informed consent was obtained from all study subjects.

### Data collection

A standardized clinical manifestation form is completed at baseline and at follow-up visits [25]. This assessment includes calculation of the modified Rodnan Skin Score [24] and a previously validated severity scale, the Medsger Severity Index [27]. Electrocardiograms, chest radiographs, and laboratory tests including complete blood count, metabolic panel, creatine phosphokinase and urinalysis are also obtained at baseline and annually thereafter. Pulmonary fibrosis on chest radiograph is defined as increased interstitial markings not explained otherwise. Information on annual income and educational levels is also collected and categorized as described in Supplement 1 in Additional file 1. Furthermore, the following autoantibodies are determined in the laboratories of the University of Texas Health Science Center at Houston: anti-nuclear antibodies and ACA by indirect immunofluorescence utilizing HEp-2 cell substrates (Antibodies Incorporated, Davis, CA, USA); ATA, anti-Ro, anti-La, anti-Smith antibody, and anti-U1-ribonucleoprotein by passive immunodiffusion against calf thymus extract (INOVA Diagnostics, San Diego, CA, USA); and anti-RNA polymerase III antibodies by ELISA (MBL, Nagoya, Japan). Anti-fibrillarin antibodies (U3-ribonucleoprotein) are determined on patients with anti-nucleolar antibodies by immunoprecipitation of the recombinant protein at the University of Calgary, as previously described [28].

The disease duration was calculated from the time of first non-Raynaud's phenomenon symptom in our study. We also utilized a second method for calculation of the disease duration, however, in which the disease onset was defined as the time of first symptom attributable to SSc (first Raynaud's phenomenon symptom or non-Raynaud's phenomenon symptom).

Chest CT, echocardiogram and right heart catheterization are not included as a routine part of the GENISOS visit, but the results of these studies are captured if obtained as part of the usual clinical care. Only a small group of subjects had undergone a chest CT or right heart catheterization at enrollment, so the results of these studies were not included in our analysis. A larger portion of patients (23.68%), however, had undergone an echocardiogram at enrollment; the predictive significance of elevated systolic pulmonary arterial pressure, defined as a right ventricular systolic pressure >40 mmHg, was therefore investigated in this subgroup of patients.

We also determined the vital status of each patient as of November 2009 by review of the National Death Index and the Social Security Death Database.

### Pulmonary function test

PFTs are performed at the three sites on the baseline visit and annually thereafter. Variables are expressed as the percentage predicted FVC, forced expiratory volume in 1 second, total lung capacity, and diffusing capacity (liters) of carbon monoxide (DLco). The predicted values are calculated according to the patient's age, height, weight, gender and ethnicity utilizing consistent reference values. The DLco percentage predicted values are also corrected for the patient's actual hemoglobin level [29]. Furthermore, all PFTs are reviewed by a pulmonologist (RME-Y-M) and studies are excluded that do not fulfill the American Thoracic Society/European Respiratory Society criteria for pulmonary function testing [30-32].

### Statistical analysis

The generalized linear mixed models were used to evaluate the effects of the measured baseline demographic and clinical variables on the percentage predicted FVC. First, the association of the baseline variables with serial measurements of percentage predicted FVC was examined. Subsequently, predictor-follow-up time interactions were included in the models to evaluate the effects of the baseline and clinical variables on the change in percentage predicted FVC over time. Those interactions evaluated whether a predictor was an effect modifier (that is, whether the change in percentage predicted FVC over time depended on the baseline variable). In other words, a baseline variable was considered a predictor of decline rate in FVC if the interaction term between the variable and the follow-up time was significant with regard to serial measurements of percentage predicted FVC.

The identification of predictors was conducted for two different follow-up periods. First, we limited our analysis to FVC measurements obtained within 3 years after enrollment. The cut-off point of 3 years was selected because this approximated to the average disease duration of 5 years in our cohort. Next, we analyzed all FVC measurements throughout the entire follow-up time. This approach was chosen was chosen to diminish the effects of survival bias because patients with rapidly progressive pulmonary disease had higher mortality (see results) and were less likely to be present in the long-term follow-up group (up to 11.4 years).

We treated patients as a sample from a larger population, and modeled between-patient variability in percentage predicted FVC as a random intercept. We also modeled between-patient variability in the change in percentage predicted FVC over time by a random slope (that is, we estimated a separate slope for each patient). We accounted for the correlations among random-effect parameters by an independent covariance matrix. Exchangeable or unstructured covariance matrices did not improve model fit evaluated by the Aikaike Information Criteria. The relationship between follow-up time and FVC was appropriately modeled by a linear function (a second-order polynomial did not improve the model fit). Generally, mixed-effect models allow inclusion of all data points in the analysis and can be used when some data points are missing.

Box-and-whisker plots were used to illustrate the trend in progression of percentage predicted FVC in the overall population and subgroups of patients based on disease type [33] and on disease duration at enrollment. *P *< 0.05 was considered significant. Analyses were conducted with Stata 10.1 statistical software (StataCorp LP, College Station, TX, USA).

## Results

### Sample characteristics

Between January 1998 and October 2009, 266 patients were enrolled in the GENISOS cohort. The mean (± standard deviation) follow-up time was 3.76 (± 3.35) years, ranging up to 11.39 years. A total of 926 FVC measurements belonging to 244 patients fulfilled the American Thoracic Society/European Respiratory Society criteria and were included in the analysis. Overall, 182 patients had at least two FVC measurements that fulfilled these criteria.

The mean (± standard deviation) age of patients at enrollment was 48.63 (± 13.5) years; 80.1% of the study population was female. The cohort consisted of 125 (47%) white, 77 (29%) Hispanic patients, 54 (20.3%) African American, and 10 (3.8%) subjects with other ethnic backgrounds. Only 49 patients (18.4%) were current smokers at the time of enrollment. Table 1 presents the demographic characteristics of patients at baseline.

**Table 1:** Baseline demographic characteristics of patients enrolled in the GENISOS cohort

Characteristic	Baseline value
Age (years)	48.63 (± 13.5)
Ethnicity	
White	125 (46.99%)
Hispanic	77 (28.95%)
African American	54 (20.3%)
Other	10 (3.76%)
Female sex	221 (83.08%)
Current smoker	49 (18.42%)
Ever smoker	134 (50.38%)
Disease duration (years)^a^	2.53 (± 1.63)
Limited skin involvement	110 (41.35%)
Autoantibodies	
Antinuclear antibodies	254 (95.49%)
Anti-centromere antibodies	32 (12.03%)
Anti-topoisomerase antibodies	49 (18.42%)
Anti-RNA polymerase III	61 (22.93%)
Anti-U1-ribonucleoprotein	30 (11.28%)
Anti-Ro	10 (3.8%)
Anti-fibrillarin	28 (10.69%)
Percentage predicted forced vital capacity	
> 80%	132 (58.15%)
50 to 80%	81 (35.68%)
< 50%	14 (6.17%)

Data presented as mean (± standard deviation) or *n *(%). ^a^Time interval between date of onset of first non-Raynaud's phenomenon symptom and enrollment.

The mean (± standard deviation) disease duration at enrollment was 2.53 (± 1.63) years; 41.4% of patients had limited cutaneous disease. ACA, ATA and anti-RNA polymerase III antibodies were present in 12.1%, 18.4% and 22.9% of patients, respectively. The proportion of patients with a baseline FVC <80% was 41.9%. Furthermore, only 22 patients (8.3%) had received cyclophosphamide before enrollment or during the follow-up period. Clinical characteristics of the GENISOS cohort at enrollment are presented in Table 1 and in Supplement 2 in Additional file 1. Moreover, a detailed comparison of baseline demographic and clinical characteristics between the investigated ethnic groups has been published previously [18].

### Factors associated with percentage predicted FVC

We next examined the association of baseline characteristics with FVC. For this analysis, we included all available baseline and follow-up FVC measurements as our outcome variable and accounted for within-subject correlation. As expected, the follow-up time was associated with a decline in percentage predicted FVC (*P *< 0.001). Furthermore, the following variables were associated with a lower percentage predicted FVC after adjustment for follow-up time (Table 2): African American ethnicity in comparison with white ethnicity (*P *= 0.002), presence of ATA (*P *< 0.001), higher modified Rodnan Skin Score (*P *= 0.001), higher visual analog score for dyspnea (*P *< 0.001), presence of basal crackles on physical examination (*P *< 0.001), signs of pulmonary fibrosis on chest radiograph (*P *< 0.001), and abnormal creatine phosphokinase (*P *= 0.046). While the presence of ACA (*P *< 0.001) and limited disease type (*P *= 0.012) were each associated with higher percentage predicted FVC. As expected, higher baseline values of the following PFT parameters correlated with higher serially measured FVC: percentage predicted FVC (*P *< 0.001), percentage predicted forced expiratory volume in 1 second (*P *< 0.001), percentage predicted DLco (*P *< 0.001), percentage total lung capacity (*P *< 0.001) and the forced expiratory volume in 1 second/FVC ratio <0.75 (*P *= 0.004).

**Table 2:** Predictive significance of baseline demographic and clinical characteristics for progression of percentage predicted FVC

Prognostic variable	Main effect adjusted for follow-up time ^d^		Interaction term of baseline FVC within 3 years of follow-up ^e^		Interaction term between baseline FVC and up to 11 years of follow-up ^e^	
			
	*b *(95% CI)	*P *value	*b *(95% CI)	*P *value	*b *(95% CI)	*P *value
Follow-up time (years)	-0.96 (-1.31 to -0.61)	**< 0.001**	NA	NA	NA	NA
Age at enrollment (years)	0.12 (-0.09 to 0.33)	0.263	-0.01 (-0.07 to 0.07)	0.914	-0.01 (-0.04 to 0.02)	0.592
Ethnicity						
White	0				0	
African American	-11.59 (-19 to -4.19)	**0.002**	0.87 (-1.5 to 3.24)	0.473	0.13(-0.87 to 1.13)	0.799
Hispanic	-0.82 (-7.24 to 5.59)	0.802	0.36 (-1.9 to 2.6)	0.752	-0.54 (-1.45 to 0.37)	0.243
Other	-10.79 (-25.42 to 3.83)	0.148	0.37 (-3.51 to 4.26)	0.851	-0.72 (-2.39 to 0.94)	0.395
Female sex	6.76 (-0.38 to 13.9)	0.063	-2.19 (-4.42 to 0.05)	0.055	-0.57 (-1.51 to 0.37)	0.233
Body mass index	-0.03 (-0.53 to 0.47)	0.905	-0.11 (-0.26 to 0.03)	0.138	0.01 (-0.06 to 0.07)	0.789
Current smoker	4.89 (-2.1 to 11.88)	0.17	0.91 (-1.16 to 2.99)	0.388	0.08 (-0.8 to 0.95)	0.867
Ever smoker	1.95 (-3.6 to 7.5)	0.491	1.25 (-0.43 to 2.93)	0.146	0.05 (-0.67 to 0.76)	0.899
Education level	1.39 (-0.13 to 2.91)	0.073	0.09 (-0.25 to 0.43)	0.599	-0.01 (-0.19 to 0.19)	0.999
Income level	0.39 (-0.93 to 1.71)	0.563	0.14 (-0.29 to 0.57)	0.523	-0.03 (-0.2 to 0.14)	0.746
Disease duration^a^	-0.26 (-1.97 to 1.45)	0.767	0.03 (-0.49 to 0.54)	0.924	-0.01 (-0.23 to 0.21)	0.94
Disease duration (alternative method)^b^	0.54 (0.04 to 1.04)	**0.034**	0.04 (-0.09 to 0.17)	0.582	-0.01 (-0.07 to 0.05)	0.753
Limited disease	7.08 (1.57 to 12.59)	**0.012**	-0.66 (-2.35 to 1.0.03)	0.442	0.03 (-0.68 to 0.74)	0.932
Autoantibodies						
ANA	-2.07 (-16.01 to 11.87)	0.771	0.46 (-3.34 to 4.27)	0.812	-1.07 (-2.74 to 0.61)	0.214
ACA	22.73 (14.82 to 30.65)	**< 0.001**	-1.74 (-4.29 to 0.81)	0.182	-0.39 (-1.43 to 0.65)	0.463
ATA	-12.49 (-19.44 to -5.53)	**< 0.001**	-2.49 (-4.62 to -0.36)	**0.022**	-0.72 (-1.63 to 0.19)	0.121
Anti-RNA polymerase III	-0.75 (-7.35 to 5.86)	0.824	0.22 (-1.69 to 2.13)	0.825	0.02 (-0.79 to 0.84)	0.955
Anti-U1-ribonucleoprotein	-1.32 (-10.18 to 7.54)	0.771	0.47 (-2.96 to 3.9)	0.788	0.08 (-1.24 to 1.4)	0.901
Anti-Ro	-1.83 (-18.42 to 14.75)	0.829	4.63 (-0.29 to 9.56)	0.065	0.89 (-1.16 to 2.94)	0.394
Anti-fibrillarin	-3.42 (-12.26 to 5.41)	0.448	-0.49 (-3.58 to 2.58)	0.751	-0.06 (-1.35 to 1.22)	0.921
Modified RSS	-0.41 (-0.65 to -0.17)	**0.001**	-0.95 (-2.37 to 0.47)	0.191	0.01 (-0.02 to 0.04)	0.43
VAS for dyspnea	-1.98 (-2.86 to -1.1)	**< 0.001**	0.21 (-0.1 to 0.52)	0.193	0.05 (-0.09 to 0.19)	0.471
VAS for pain	-0.35 (-0.91 to 0.21)	0.221	0.07 (-0.07 to 0.22)	0.331	0.05 (-0.02 to 0.12)	0.184
Teleagiectasia						
Facial/oral	2.78 (-2.76 to 8.31)	0.326	1.24 (-0.44 to 2.91)	0.148	0.35 (-0.35 to 1.05)	0.329
Palmar	1.34 (-4.65 to 7.33)	0.661	1.09 (-0.68 to 2.88)	0.225	0.51 (-0.22 to 1.25)	0.176
Dysphagia	1.39 (-4.14 to 6.93)	0.621	0.13 (-1.55 to 1.82)	0.879	0.3 (-0.41 to 1.01)	0.404
sPAP increase by echo	0.2 (-13.36 to 13.75)	0.977	3.51 (0.27 to 6.74)	**0.033**	0.16 (-0.40 to 3.51)	0.119
Auscultatory rales	-16.64 (-22.92 to -10.36)	**< 0.001**	0.45 (-1.49 to 2.39)	0.651	-0.32 (-1.17 to 0.54)	0.464
Fibrosis on CXR	-26.67 (-33.07 to -20.27)	**< 0.001**	1.09 (-1.35 to 3.54)	0.379	-0.8 (-1.85 to 0.25)	0.137
Baseline FVC%^c^	0.94 (0.86 to 1.02)	**< 0.001**	-0.01 (-0.07 to 0.06)	0.869	-0.02 (-0.4 to 0.01)	0.066
Baseline FEV1%	0.94 (0.87 to 1.01)	**< 0.001**	0.01 (-0.07 to 0.08)	0.874	-0.01(-0.03 to 0.01)	0.222
Baseline DLco%	0.46 (0.36 to 0.57)	**< 0.001**	-0.01 (-0.01 to 0.01)	0.387	0.01 (-0.01 to 0.02)	0.655
Baseline TLC%	0.82 (0.73 to 0.92)	**< 0.001**	0.01 (-0.01 to 0.01)	0.175	-0.01 (-0.03 to 0.01)	0.389
FEV1/FVC <0.75	9.16 (2.88 to 15.45)	**0.004**	-1.52 (-5.98 to 2.94)	0.505	-0.21 (-0.98 to 0.56)	0.597
Creatinine ≥1.5	-6.91 (-24.89 to 11.07)	0.452	4.88 (-1.35 to 11.11)	0.125	-0.35 (-3.52 to 2.82)	0.828
CPK >200	-7.81 (-15.5 to -0.13)	**0.046**	2.1 (-0.26 to 4.47)	0.081	0.49 (-0.52 to 1.51)	0.337
Hematocrit	-0.01 (-0.64 to 0.64)	0.999	-2.75 (-11.14 to 5.65)	0.521	-0.02 (-0.11 to 0.07)	0.685
White blood cells	-0.91 (-1.89 to 0.06)	0.067	-0.01 (-0.01 to 0.01)	0.072	0.04 (-0.07 to 0.16)	0.465
Elevated platelet count	-3.37 (-9.62 to 2.86)	0.289	0.08 (-1.64 to 1.8)	0.928	-0.22 (-1.03 to 0.59)	0.599

ACA, anti-centromere antibodies; ANA, anti-nuclear antibodies; ATA, anti-topoisomerase antibodies; *b*, regression coefficient; CI, confidence interval; CPK, creatine phosphokinase; CXR, Chest X-ray; DLco, lung diffusion capacity for carbon monoxide; FEV1, forced expiratory volume in 1 second; FVC, forced vital capacity; RSS, Rodnan Skin Score; sPAP, systolic pulmonary arterial pressure; TLC, total lung capacity; VAS, visual analog scale. ^a^Disease onset defined as first non-Raynaud's phenomenon symptom. ^b^Disease onset defined as first symptom attributable to SSc. ^c^Baseline FVC measurements were not included in the outcome variable for this analysis. ^d^Main effect adjusted for follow-up time. ^e^Interaction term between the follow-up time and the respective baseline variable (outcome: rate of decline in FVC). Bold data represent significant associations (*P *< 0.05).

As shown in Table 2, the presence of anti-RNA polymerase III, anti-fibrillarin and anti-U1-ribonucleoprotein antibodies, the body mass index and other examined demographic and clinical variables were not significantly associated with percentage predicted FVC.

We also examined the relationship of subscales of the Medsger Severity Index with percentage predicted FVC. As shown in Table 3, higher scores (more severe) for the skin (*P *= 0.037) and lung (*P *< 0.001) subscales were associated with lower levels of the investigated outcome variable, whereas the other subscales (general, peripheral vascular, joint/tendon, gastrointestinal tract, heart and kidney) showed no significant relationship to FVC.

**Table 3:** Predictive significance of Severity Index subscales for progression of FVC

Medsger Severity Index	Main effect adjusted for follow-up time		Interaction term of baseline FVC within 3 years of follow-up		Interaction term between baseline FVC and up to 11 years of follow-up	
			
	*b *(95% CI)	*P *value	*b *(95% CI)	*P *value	*b *(95% CI)	*P *value
General	-0.22 (-3.65 to 3.19)	0.897	0.0 (-0.01 to 0.01)	0.463	0.01 (-0.48 to 0.5)	0.972
Peripheral vascular	-0.81 (-3.31 to 1.69)	0.525	0.0 (-0.01 to 0.01)	0.436	0.1 (-0.21 to 0.41)	0.513
Skin	-3.61 (-7 to -0.22)	**0.037**	0.58 (-2.41 to 1.24)	0.531	0.17 (-0.27 to 0.61)	0.449
Joint/tendon	-2.14 (-4.48 to 0.19)	0.072	-0.19 (-1.15 to 0.75)	0.682	-0.06 (-0.37 to 0.25)	0.712
Muscle	-5.61 (-12.22 to 1)	0.096	-0.32 (-1.27 to 0.63)	0.504	0.23 (-0.61 to 1.07)	0.588
Gastrointestinal tract	-0.26 (-4.58 to 4.06)	0.905	0.0 (-0.01 to 0.01)	0.369	0.02 (-0.61 to 0.65)	0.955
Lung	-9.73 (-11.91 to -7.55)	**< 0.001**	0.0 (-0.01 to 0.01)	0.707	-0.29 (-0.62 to 0.03)	0.077
Heart	-1.98 (-6.17 to 2.2)	0.353	0.0 (-0.01 to 0.01)	0.321	0.24 (-0.44 to 0.92)	0.493
Kidney	0.62 (-6.84 to 8.09)	0.87	0.0 (-0.01 to 0.01)	0.128	-0.22 (-1.64 to 1.19)	0.756

*b*, regression coefficient; CI, confidence interval; FVC, forced vital capacity. Bold data represent significant associations (*P *< 0.05).

In addition, the smoking status did not show a significant relationship to FVC. Furthermore, none of the above-mentioned associations changed after correction for current smoking status. Similarly, the above-mentioned associations did not change after exclusion of patients ever treated with cyclophosphamide (data not shown).

### Role of ethnicity in progression of restrictive lung disease in systemic sclerosis

We have previously reported that African American patients with SSc had a higher frequency of pulmonary fibrosis and a lower percentage predicted FVC at the baseline visit in the GENISOS cohort [18]. As demonstrated in Table 2, this group of patients also had lower percentage predicted FVC after inclusion of all FVC measurements, whereas this outcome variable among Hispanic patients did not differ from that for white patients. In a multivariable model, percentage predicted FVC among the African American patients remained significantly lower than white patients even after adjustment for disease duration, ATA and ACA positivity, disease type, and smoking status, in addition to income and education levels (*P *= 0.038; regression coefficient (RC) = -7.73, confidence interval (CI) = -15.02 to -0.45), whereas Hispanic patients did not differ from white patients (*P *= 0.733; RC = 1.21, CI = -5.33 to 7.58). These results indicate that African American patients had lower percentage predicted FVC independent of smoking status, socioeconomic factors, disease duration/type (limited versus diffuse cutaneous disease) and serology.

### Predictors of rate of decline in percentage predicted FVC

**Figure 1:**
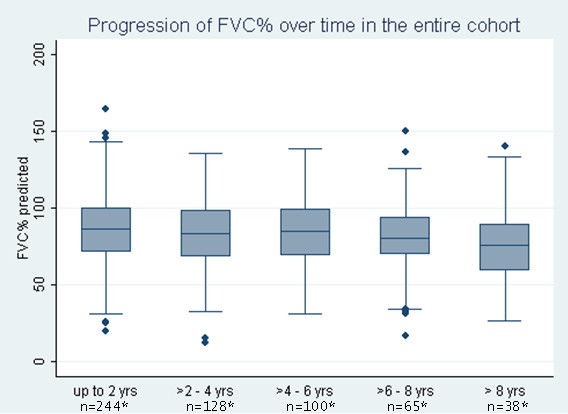
**Course of percentage predicted forced vital capacity over 2-year intervals of follow-up**. Percentage predicted forced vital capacity (FVC%) data presented in box-and-whisker plots. Each box represents the 25th to 75th percentile: length of box represents interquartile range (IQR); line inside represents median. Whiskers represent 1.5 times the upper and lower IQRs. Circles indicate outliers. *Number of patients who had at least one FVC measurement during the follow-up interval.

As shown in Figure 1, our data did not indicate that the overall decline in percentage predicted FVC slowed down during the follow-up time. This was also demonstrated by the fact that the curvilinear model for progression of percentage predicted FVC did not show a better fit to the data than the linear model. The frequency of patients with ≥10% decline in percentage predicted FVC increased at a slower pace, however, from 16.7% in the first year of follow-up to 45.9% after ≥8 years of follow-up (Table 4).

**Table 4:** Frequency of 5% or 10% decline in percentage predicted FVC during the follow-up period

Frequency of decline in baseline FVC	Follow-up year							
	
	1	2	3	4	5	6	7	> 8
≥5%	31.2	36.4	41.0	49.3	47.9	52.5	46.2	51.4
≥10%	16.7	18.2	23.1	31.0	32.9	30.5	38.5	45.9

We next investigated which baseline variables are predictors of accelerated decline in FVC. For this purpose, the interaction term between the respective variable and follow-up time was examined. As shown in Tables 2 and 3, presence of ATA was the only variable associated with sequentially obtained FVC levels that also predicted the rate of decline within the first 3 years of follow-up. Presence of ATA was associated with a higher decline rate in FVC in this time period (*P *= 0.022, RC = -2.49, CI = -4.62 to -0.36). Presence of ATA was not a significant predictor of FVC decline rate, however, when the follow-up time was not restricted (*P *= 0.121). The latter analysis included FVC measurements that were obtained up to 11.4 years after enrollment.

The interaction term for elevated systolic pulmonary arterial pressure on the echocardiogram was also significant when the analysis was restricted to 3-year follow-up. However, this variable was not associated with sequentially obtained FVC levels (insignificant main effect, *P *= 0.997). Furthermore, an echocardiogram was obtained only in 23.7% of patients at enrollment.

Other baseline variables associated with differential FVC levels did not predict progression of this variable over time in 3-year or long-term follow-up analyses. Specifically, ethnicity, autoantibody status, subjective dyspnea, baseline PFT values, Medsger Severity Index subscores and smoking status did not predict the rate of progression in FVC.

**Figure 2:**
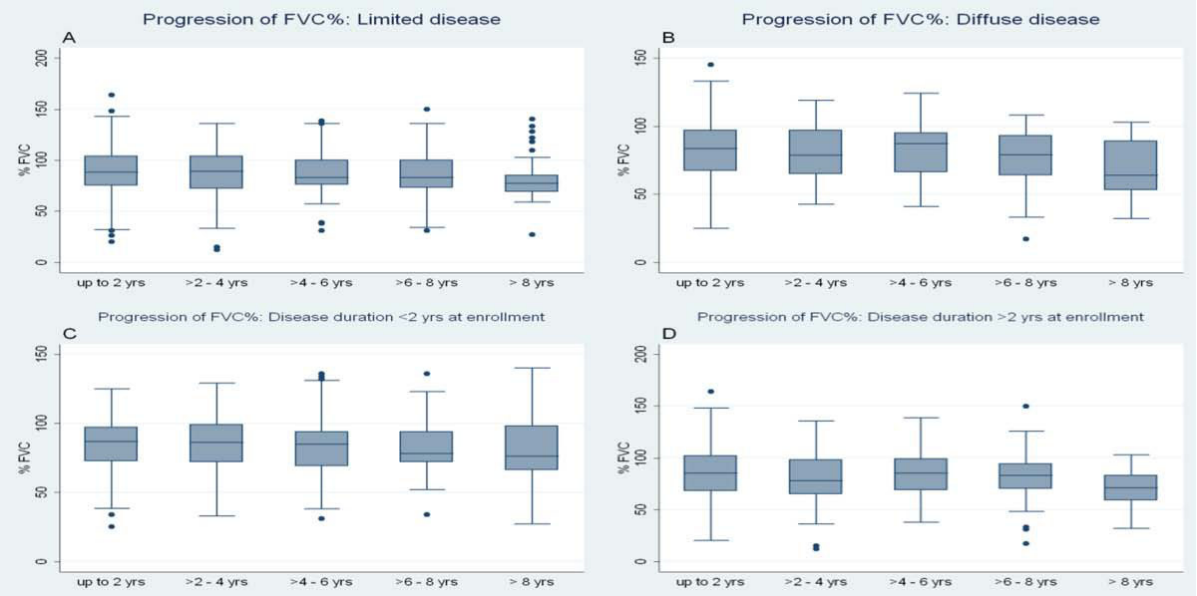
**Progression of percentage predicted forced vital capacity**. Percentage predicted forced vital capacity (FVC%) progression in **(a) **patients with limited cutaneous involvement, **(b) **patients with diffuse cutaneous involvement, **(c) **patients with disease duration at enrollment <2 years, and **(d) **patients with disease duration at enrollment >2 years. Data are shown as box-and-whisker plots; for more details, see Figure 1.

Figure 2a,b shows graphically that the progression of the outcome variable was not significantly different among patients with limited versus diffuse disease types; this was further supported by the fact that the interaction term between disease type and follow-up time was not significant (*P *= 0.442 for 3-year follow-up, *P *= 0.932 for long-term follow-up). Similarly, the interaction term for disease duration at enrollment and follow-up time also was not significant (*P *= 0.767 for 3-year follow-up, *P *= 0.94 for long-term follow-up), indicating that shorter disease duration did not predicted a significantly faster rate of decline in FVC. To demonstrate this observation graphically, SSc patients were divided into two groups based on disease duration of 2 years. One hundred and nineteen (44.7%) patients had disease duration <2 years at the initial visit. As indicated in Figure 2c,d, the slope of decline in the percentage predicted FVC was not significantly different between these two groups.

Similarly, the disease duration measured from the time of first symptom attributable to SSc (see Table 2, Disease duration (alternative method)) did not predict the rate of decline in the percentage predicted FVC (*P *= 0.582 for 3-year follow-up, *P *= 0.753 for long-term follow-up.

### Rate of FVC decline among deceased patients

Seventy-three patients (27.4%) died during follow-up. As expected, the deceased patients had lower serial FVC values (*P *= 0.001, RC = -10.08, CI = -16.26 to -3.89). Furthermore, the examination of the interaction term between follow-up time and vital status revealed that the deceased patients had a significantly faster rate of decline in percentage predicted FVC compared with the remainder of the patients (*P *= 0.001, RC = -1.66, CI = -2.63 to -0.69) for long-term follow-up).

## Discussion

In the current study, we investigated the association and predictive significance of baseline demographic and clinical variables with sequentially obtained percentage predicted FVC measurements in a large prospective cohort of early SSc patients. Similar to our previous cross-sectional study [18], a number of baseline characteristics were associated with FVC levels. Only ATA, however, was associated with lower serial FVC levels as well as with a higher decline rate in FVC at 3-year follow-up.

Our results confirm the previously reported association of ACA [11-15,34] and ATA with FVC levels; that is, those patients with ACA have higher percentage predicted FVC, and those patients with ATA have lower percentage predicted FVC. Previous reports also have indicated that patients with anti-RNA polymerase III antibodies have extensive skin involvement but less frequent ILD [13,35]. These antibodies, however, were not associated with higher percentage predicted FVC levels in our study. Presence of ATA was also a predictor of an accelerated decline rate in FVC within 3 years of enrollment; however, presence of ATA was not a significant predictor of the FVC decline rate on long-term follow-up (up to 11.5 years). This discrepancy can be partially explained by the fact that patients with rapidly progressive ILD had higher mortality and were not well represented in the long-term follow-up group.

The GENISOS cohort includes a considerable number of Hispanic patients (*n *= 77) and African American patients (*n *= 54), raising the possibility that our findings also are generalizable to nonwhite patient populations. In our study, African American patients had lower levels of percentage predicted FVC, even after adjusting for socioeconomic factors, antibody status and smoking habits. This observation might partially explain lower survival of nonwhite patients in the GENISOS cohort [36] and in other studies [37-39]. However, our results confirmed that African American ethnicity *per se *does not predict the rate of decline in percentage predicted FVC [7,20].

One study reported that SSc patients who were former smokers had an accelerated rate of decline in FVC [19], but this finding has not been confirmed by other studies [7,14,20,22]. We did not observe an association between smoking and percentage predicted FVC levels or its rate of decline. It is possible that smoking status influences progression of other PFT parameters such as the total lung capacity and DLco.

Two retrospective studies indicated that low baseline FVC levels predicted accelerated rate of decline in this variable over time [16,22]. Both studies conducted a time to decline analysis rather than directly measuring the decline rate in FVC. Other studies, using an annualized rate of decline to measure percentage predicted FVC, reported no association between baseline FVC and the annualized rate of decline [7,14,21]. Similarly, our study did not indicate that low baseline PFT parameters predicted a higher rate of decline in FVC on subsequent visits.

Some studies have indicated that the loss of volume on PFTs was greatest early in the course of disease and that the rate of decline in percentage predicted FVC decelerates over time [7,8,20], while others could not confirm this finding [9]. Disease onset was defined as the time of first symptom attributable to SSc in the first two studies [7,14]. In our study, the overall rate of decline in percentage predicted FVC did not slow down during the follow-up time; similar results were seen in our subgroup analyses according to disease type and duration. We used the two most commonly adopted methods for calculation of disease duration in our study. The disease duration determined by both calculation methods was not a significant predictor of rate of decline in FVC. Similar to our findings, the rate of decline in FVC did not differ significantly in the Scleroderma Lung Study when patients were stratified according to disease duration [40]. The frequency of patients with ≥10% decline in FVC, however, showed its highest increase in the first follow-up year and increased only at a slower pace to 45.9% after ≥8 years of follow-up in our study (Table 4). This raises the possibility that a smaller subgroup of patients has an accelerated decline rate in the initial phase of disease. The FVC decline in these patients might stabilize in later follow-up years if they survive the initial rapid worsening of the ILD. However, this subgroup might not be well represented in patients with longer follow-up time because of its higher mortality rate.

We utilized a random-effect repeated-measurement model for our analyses. This approach utilizes all follow-up FVC measurements and accounts for the within-subject correlation. The approach therefore provides more accurate estimation of predictors for rate of decline than the time to decline or the annualized rate of decline analysis methods utilized in previous studies [7,14,16,17,19-21,23].

Confirming findings of a previous study [21], we observed that a faster rate of decline in percentage predicted FVC was a marker of poor survival. This finding emphasizes the importance of predictive parameters for progression of FVC. However, our findings also support the results of previous studies [7,14,20,21,23] in which routinely obtained demographic and clinical parameters - except for ATA - failed to predict the rate of decline in FVC over time.

Consequently, our study provides further support for the identification of predictive biomarkers utilizing novel imagining and molecular techniques. Two previous reports indicated that the extent of reticular pattern on high-resolution computed tomography (HRCT) utilizing a semiquantitative scoring system predicts further decline in FVC [22,41]. The fibrosis score based on HRCT was not captured in our study and is not yet available for routine clinical use, but has promise for future studies. Another study has indicated that high sedimentation rates at baseline predict a faster decline in percentage predicted FVC [14]. Furthermore, SSc patients with ILD had more severe reflux disease on monometry and impedance PH monitoring in a cross-sectional study [42]. The severity of esophageal involvement on monometry, however, was not predictive of the decline in FVC in a prospective study [17].

Furthermore, a polymorphism in the *IRF5 *gene was associated with ILD in a cross-sectional sample of SSc patients [43]. Others have reported that pneumoproteins such as surfactant protein D and Krebs von den Lungen-6 correlate with the presence of pulmonary fibrosis and alveolitis in SSc [44,45]. However, large observational cohort studies with a collection of genetic data and serial blood samples are needed to investigate the predictive significance of these potentially useful biomarkers.

Cyclophosphamide is the only treatment for ILD that has been demonstrated to be effective in SSc [41,46]. Considering that only a small portion of our patients were ever treated with cyclophophamide (8.27%), we believe our observations are close to the natural history of ILD in SSc. Furthermore, a subgroup analysis after exclusion of patients ever treated with cyclophosphamide did not change our overall results. We cannot, however, exclude that treatment with various potentially effective medications may have influenced our findings. Another limitation of the current study is that the majority of investigated patients did not undergo HRCT at enrollment, and therefore the predictive significance of the previously described semiquantitative scoring system on HRCT could not be investigated in our cohort [22,41]. Furthermore, most of our patients were recruited from tertiary-care rheumatology clinics in South Texas, raising the possibility of a referral bias toward more severe disease.

Despite the present study's prospective design and relatively large sample size, we cannot exclude the possibility that we are still underpowered to identify more subtle clinical and demographic predictors of decline in percentage predicted FVC. Furthermore, it is possible that some of the observed associations are false-positive findings; we did not correct for multiple comparisons in order to decrease the likelihood of missing clinically important associations (β-error).

## Conclusions

Our results support a continuous loss of pulmonary vital capacity that persists into the later course of SSc. A number of baseline variables were associated with serially measured levels of percentage predicted FVC. Presence of ATA was the only variable associated with serial FVC measurements that also predicted the rate of decline in FVC within the first 3 years of follow-up. None of the above-mentioned variables, however, predicted the decline rate in vital capacity upon longer follow-up time (up to 11.4 years). A faster rate of decline in percentage predicted FVC was associated with poor survival, emphasizing the need for identification of predictive biomarkers. Large observational studies with collection of genetic data and serial blood samples are an important asset for identification of new predictors.

## Abbreviations

ACA: anti-centromere antibodies; ATA: anti-topoisomerase I antibodies; CI: confidence interval; CT: computed tomography; DLco: diffusing capacity of carbon monoxide; ELISA: enzyme-linked immunosorbent assay; FVC: forced vital capacity; GENISOS: Genetics versus Environment in Scleroderma Outcome Study; HRCT: high-resolution computed tomography; ILD: interstitial lung disease; PFT: pulmonary function test; RC: regression coefficient; SSc: systemic sclerosis.

## Competing interests

MJF is a paid consultant to ImmunoConcepts Inc., a manufacturer of autoantibody diagnostic kits. The remaining authors declare that they have no competing interests.

## Authors' contributions

SA, RS, TAM, RME-Y-M, HD, MJF, JDR, FCA, MDM contributed to the study design, data acquisition and drafting of the manuscript. REL and DKN contributed to the study design, data analysis and drafting of the manuscript. All authors reviewed, edited and approved the final version of the manuscript.

## Supplementary Material

Additional file 1 **Supplements 1 & 2**. Supplement 1: Scoring systems for annual household income and educational level. Explanation of the scoring system utilized for capturing the annual income and educational level of patients enrolled in the GENISOS cohort. Supplement 2: Baseline clinical characteristics. The baseline clinical characteristics of patients enrolled in the GENISOS cohort. Click here for file
