# Supplementary material for: Predictors of interstitial lung disease in early systemic sclerosis: a prospective longitudinal study of the GENISOS cohort
Source: Arthritis Res Ther. 2010 Sep 2;12(5):R166. doi: 10.1186/ar3125 (PMC2990992; doi:10.1186/ar3125)
Supplement: Additional file 1 — Supplements 1 & 2. Supplement 1: Scoring systems for annual household income and educational level. Explanation of the scoring system utilized for capturing the annual income and educational level of patients enrolled in the GENISOS cohort. Supplement 2: Baseline clinical characteristics. The baseline clinical characteristics of patients enrolled in the GENISOS cohort. [file ar3125-S1.DOC]

Supplement 1:

Scoring System for Annual Household Income:

1 = < $5,000

2= $5,000 - 9,999

3=$ 10,000 - 14,999

4= $15,000 - 19,999

5= $ 20,000 - 29,999

6 = $ 30,000 - 49,999

7 = $ 50,000 - 99,999

8 = >=$ 100,000

Scoring System for Educational Level:

Less than High School Graduate 🡪0

High School Diploma or Equivalent🡪1

Associate Degree 🡪2

Bachelor’s Degree🡪3

Master’s Degree 🡪4

Doctorate 🡪5

Professional (MD, JD, DDS, etc.) 🡪 6

Supplement 2:

Table 1: Baseline clinical characteristics of patients enrolled in the GENISOS*

| Characteristics |  |
| --- | --- |
| MRSS , mean, SD | 15.8 (11.75) |
| Disease duration (alternative method) †, mean, SD | 4.68 (0.16) |
| Visual analogue score for dyspnea, median | 1.3 |
| Visual analogue score for pain, median | 4.05 |
| Telenagiectasia  Facial/oral  Palmar |  |
|  | 118 (44.36) |
|  | 81 (30.45) |
| Dysphagia, n, % | 131 (49.25) |
| PAH by echocardiogram‡, n, % | 16 (23.9) |
| Crackles on physical examination, n, % | 63 (23.68) |
| Pulmonary fibrosis on CXR, n, % | 49 (18.42) |
| FVC % predicted, mean, SD | 84.15 (22.16) |
| FEV1% predicted, mean, SD | 81.87 (20.68) |
| DLco% predicted, mean, SD | 70.72 (23.28) |
| TLC% predicted , mean, SD | 91.26 (20.94) |
| Creatinine ≥ 1.5,n, % | 7 (2.63) |
| CPK>200, n, % | 39 (14.67) |
| Hematocrit, mean, SD | 38.2 (4.39) |
| White Blood Cell Count, mean, SD | 7.6 (2.81) |
| Platelet Count, mean, SD | 286.37 (93.65) |
| Medsger Severity Index |  |
| General, mean, SD | 0.46 (0.81) |
| Peripheral vascular, mean, SD | 1.53 (1.1) |
| Skin, mean, SD | 1.57(0.83) |
| Joint/tendon, mean, SD | 0.7(1.23) |
| Muscle, mean, SD | 0.21(0.42) |
| GI Tract, mean, SD | 0.7(0.64) |
| Lung, mean, SD | 1.34(1.12) |
| Heart, mean, SD | 0.27(0.73) |
| Kidney, mean, SD | 0.09(0.44) |

*MRSS= Modified Rodnan Skin Score; COPD= Chronic obstructive pulmonary disease; PAH= Pulmonary Arterial Hypertension; FVC= Forced vital capacity; FEV1=Forced expiratory volume in 1 second; DLco= lung diffusion capacity for Carbon monoxide; TLC= Total lung capacity; CPK= Creatine Phosphokinase.

†Disease onset defined as first symptom attributable to SSc

‡Baseline echocardiogram results were available in 63 patients
